# Perioperative CRP: A novel inflammation‐based classification in gastric cancer for recurrence and chemotherapy benefit

**DOI:** 10.1002/cam4.3514

**Published:** 2020-12-03

**Authors:** Jun Lu, Bin‐Bin Xu, Zhen Xue, Jian‐Wei Xie, Chao‐Hui Zheng, Chang‐Ming Huang, Ping Li

**Affiliations:** ^1^ Department of Gastric Surgery Fujian Medical University Union Hospital Fuzhou China; ^2^ Department of General Surgery Fujian Medical University Union Hospital Fuzhou China; ^3^ Key Laboratory of Ministry of Education of Gastrointestinal Cancer Fujian Medical University Fuzhou Fujian Province China

**Keywords:** adjuvant chemotherapy, C‐reactive protein, gastric cancer, prognosis, recurrence

## Abstract

**Background:**

Perioperative C‐reactive protein (CRP) levels have effects on the prognosis of cancer patients. We intended to determine the prognostic value of combining the two for gastric cancer (GC).

**Methods:**

Data were extracted from a clinical trial. By calculating the area under the curve (AUC) and the C‐index, the predictive value of CRPs among different time points, including preoperative (pre‐CRP), postoperative days 1, 3, and 5 (post‐CRPs), and postoperative maximum CRP (post‐CRP_max_), was derived. Multivariate analysis was performed to further explore the independent variates for recurrence‐free survival (RFS).

**Results:**

Finally, 401 patients were available in the present study. For RFS, higher AUC (0.692) and concordance index (0.678) of pre‐CRP were observed when compared with those of post‐CRPs. Further, among post‐CRPs, post‐CRP_max_ had the highest predictive values (AUC: 0.591; concordance index: 0.585) among the other post‐CRPs. The threshold values in predicting RFS for pre‐CRP and post‐CRP_max_ were 3.1 mg/L and 77.1 mg/L. Multivariate analysis showed both pre‐CRP≥3.1 mg/L (high‐pre‐CRP) and post‐CRP_max_≥77.1 mg/L (high‐post‐CRP_max_) were risk factors for RFS. Postoperative chemotherapy benefit was further analyzed for patients with stage II/III GC and indicated that patients with pre‐CRP<3.1 mg/L had better prognosis without benefit from postoperative adjuvant chemotherapy (ACT), *p* = 0.557. In high‐pre‐CRP patients, only patients with post‐CRP_max_≥77.1 mg/L but not post‐CRP_max_<77.1 mg/L benefited from postoperative ACT (RFS: 33.2% vs 49.9% for non‐chemotherapy group and chemotherapy group, respectively, *p* = 0.037). Analyses for overall survival obtained the similar outcomes.

**Conclusions:**

Both high‐pre‐CRP and high‐post‐CRP_max_ are associated with worse prognosis in GC. ACT seems to only improve the prognosis for stage II/III GC with pre‐CRP≥3.1 mg/L and post‐CRP_max_≥77.1 mg/L after radical gastrectomy. Further studies are needed to confirm these findings and explore the potential mechanism.

## Graphical abstract

Using randomized phase III trial data, we found that both high pre‐CRP and high post‐CRPmax statuses were clinically actionable as potential prognostic indicators in gastric cancer. More impressively, adjuvant chemotherapy seems to only improve the prognosis for stage II/III gastric cancer with pre‐CRP ≥3.1 mg/L and post‐CRPmax ≥77.1 mg/L.

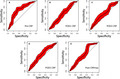

## 1 INTRODUCTION

Gastric carcinoma (GC) is the fifth most common malignancy worldwide and ranks third in cancer‐related mortality^1^ and postoperative recurrence remains common for patients after radical gastrectomy, affecting approximately 18% to 45.5% of patients.^2^, ^3^, ^4^, ^5^ Postoperative adjuvant chemotherapy (ACT) has been confirmed to improve the recurrence‐free survival (RFS) after GC surgery.^6^, ^7^, ^8^ However, there is still a lack of simple and practical classifiers that can effectively identify subgroups benefiting from ACT.

Since the correlation between inflammation and cancer was uncovered,^9^ increasingly more scholars have carried out studies on inflammatory factors and tumors.^10^ A succession of studies confirmed that patients with a high level of preoperative C‐reactive protein (CRP) (pre‐CRP) were closely related to a poor prognosis for GC,^11^, ^12^ indicating that pre‐CRP is expected to become an effective forecasting tool alone or in combination with other tumor characteristics or inflammation indices to predict the prognosis of GC. In addition, existing research shows that both pre‐CRP and postoperative CRP (post‐CRP) levels are associated with the prognoses for cancer patients.^13^, ^14^ However, the prognostic value of combining these two variables has not been reported yet, especially for GC.

Therefore, this study used prospective clinical trial data for the first time to explore whether combined pre‐CRP and post‐CRP levels can be an effective predictor of postoperative recurrence. We also explored their relationship with the efficacy of ACT.

## 2 MATERIALS AND METHODS

### 2.1 Patients

From 1 January 2015 to 1 April 2016, 438 patients were recruited to the clinical trial.^15^ The details about the inclusion criteria and exclusion criteria, were previously reported^15^ and shown in Table S1. The final analysis of the randomized controlled trial (RCT) included 419 patients. The RCT was conducted in accordance with the protocol that was approved by the institutional review boards of Fujian Medical University Union Hospital (FMUUH) (ClinicalTrials.gov number NCT02327481).^15^ The present study was also approved by the institutional review boards of FMUUH (IRB number: 2020KY0106). The present study was a subgroup analysis of the previously conducted RCT.^5^ After excluding 10 patients with neuroendocrine carcinoma, six patients with palliative surgery and two patients without evidence for GC, the present analysis was restricted to 401 patients with pathologic stage I, II, or III gastric adenocarcinoma (pT1‐4aN0‐3M0) according to the 7th American Joint Committee on Cancer tumor‐node‐metastasis (TNM) staging system, as previously described.^16^, ^17^ Patients in stage I were excluded from a subset analysis assessing the benefits of ACT.

### 2.2 Pre‐ and postoperative crp levels

We obtained preoperative serum CRP values within the 7 days prior to surgery and postoperative days (POD) 1, 3, and 5 from the patients’ records. All the patients had CRP levels tested at least once preoperatively and postoperatively. Post‐CRP_max_ was defined as the postoperative maximum CRP value from surgery until hospital discharge in the present study.

### 2.3 Adjuvant chemotherapy

According to the patient's wishes and physical condition, fluoride‐based ACT was recommended for most patients with pathological stage II and III in our center.^18^ A combination of 5‐fluorouracil and either cisplatin/oxaliplatin or paclitaxel regimens was routinely recommended in our center as previously described.^5^ Detailed, 256 patients (63.8%) received 5‐FU ACT with a median cycle of 5 (range 1–12), similar to a previous study.^19^ Specifically, 76.2% (195/256), 12.1% (31/256), 9.4% (24/256), and 2.3% (6/256) received the SOX regimen, the FOLFOX regimen, the S‐1 + paclitaxel regimen, and the S1 + docetaxel regimen, respectively.^20^ No patients received radiotherapy in the present study.

### 2.4 Follow‐up

The median follow‐up time was 42 months (range 3‐51 months) in this cohort as of the data cut‐off date (April 2019, at least 3 years after enrollment of the last patient). All surviving patients were followed‐up at least 3 years. Postoperative follow‐ups were routinely performed every 3 months for 2 years and then every 6 months from years 3 to 5. Recurrence was diagnosed based on the radiologic findings or the biopsies with suspicious lesions when possible, as previously described^5^.

### 2.5 Statistical analyses

Categorical variables were compared using the χ^2^ or Fisher's exact test and continuous variables by *t* test. Receiver operating characteristic (ROC) curves were established to estimate the optimal cut‐off values for preoperative and postoperative CRP levels as risk factors for recurrence. By calculating the area under the curve (AUC) and the C‐index, the discriminative ability of CRPs during different periods was compared. RFS was assessed using the Kaplan‐Meier method. The AIC was performed to compare the prognostic value of the different models.^21^. Factors related to RFS were identified by the Cox proportional hazards regression model. To explore that whether the postoperative complication affected the relationship between postoperative CRP_max_ and RFS, we included in our primary Cox model an additional interaction term^22^ between postoperative complication and postoperative CRP_max_ (Table 2). Decision curve analysis was also performed, which could evaluate the clinical usefulness of a prediction model by calculating its net benefit using the rate of true and false positives in varied risk thresholds for screening.^23^ All the statistical analyses were performed using SPSS v.18.0 for Windows (SPSS Inc.) and R (https://www.r‐proje ct.org/).

## 3 RESULTS

### 3.1 Clinicopathological characteristics

Table 1 showed the baseline characteristics of the cohorts. A total of 109 (27.2%) patients experienced recurrence after radical gastrectomy. The CRP levels for the recurrence patients were higher than those without recurrence at all time points, but significant differences were only observed for preoperative, POD5, and post‐CRP_max_ (all *p* < 0.05). Further, patients with recurrence were associated with poor clinical features, and all the p‐values were less than 0.05. The median OS and RFS for the whole cohorts have not been reached. The 3‐year RFS and OS rate were 72.9% (95%CI: 68.6%‐77.2%) and 77.3% (95%CI: 73.2%‐81.4%), respectively (Figure S1A,B). Figure S2 shows the CRP values at different time points before and after surgery.

**TABLE 1:** Clinicopathological characteristics of all patients

	All patients (n = 401)	No recurrence (n = 292)	Recurrence (n = 109)	*p* value
Age, mean years (SD)	58.6 (10.3)	57.9 (10.6)	60.5 (9.2)	0.018
Sex n (%)				0.298
Male	271 (67.6%)	193 (66.1%)	78 (71.6%)	
Female	130 (32.4%)	99 (66.1%)	31 (28.4%)	
Tumor diameter, mean mm (SD)	41.5 (23.1)	35.8 (21.5)	56.5 (20.3)	<0.001
Tumor location n (%)				0.158
Upper	117 (29.2%)	76 (26.0%)	41 (37.6%)	
Middle	69 (17.2%)	53 (18.2%)	16 (14.7%)	
Lower	191 (47.6%)	145 (49.7%)	46 (42.2%)	
Mix	24 (6.0%)	18 (6.2%)	6 (5.5%)	
Pathological type n (%)				<0.001
Differentiated	168 (41.9%)	138 (47.3%)	30 (27.5%)	
Undifferentiated	233 (58.1%)	154 (52.7%)	79 (72.5%)	
Lymphovascular invasion n (%)				<0.001
Absent	228 (56.9%)	205 (70.2%)	23 (21.1%)	
Present	173 (43.1%)	87 (29.8%)	86 (78.9%)	
Preop CRP (mg/L)				
Median (IQR)	3.0 (1.8‐5.1)	2.7 (1.7‐4.1)	4.8 (2.6‐6.5)	<0.001
CRP on POD1 (mg/L)^a^				
Median (IQR)	34.0 (21.7‐58.5)	32.8 (21.2‐57.3)	39.0 (22.7‐61.4)	0.168
CRP on POD3 (mg/L)^b^				
Median (IQR)	107.0(74.7‐141.3)	105.0 (72.7‐136.0)	115.0 (77.8‐149.0)	0.130
CRP on POD5 (mg/L)^c^				
Median (IQR)	63.9(40.2‐90.9)	62.4 (37.4‐86.9)	67.9 (50.3‐100.2)	0.042
Postop CRP_max_ (mg/L)				
Median (IQR)	110.0 (75.8‐143.5)	107.0 (71.5‐137.5)	118.0 (82.1‐150.0)	0.011
Postop complication n (%)				0.505
Absent	339 (84.5%)	249 (85.3%)	90 (82.6%)	
Present	62 (15.5%)	43 (14.7%)	19 (17.6%)	
Adjuvant chemotherapy n (%)				<0.001
Absent	145 (36.2%)	122 (41.8%)	23 (21.1%)	
Present	256 (63.8%)	170 (58.2%)	86 (78.9%)	
pTNM stage n (%)				<0.001
Ⅰ	135 (33.7%)	132 (45.2%)	3 (2.8%)	
Ⅱ	84 (20.9%)	77 (26.4%)	7 (6.4%)	
Ⅲ	182 (45.4%)	83 (28.4%)	99 (90.8%)	
Death n (%)				<0.001
No	323 (80.5%)	284 (97.3%)	18 (16.5%)	
Yes	78 (19.5%)	8 (2.7%)	91 (83.5%)	

Abbreviations: CRP_max_, maximum CRP value; CRP, C‐reactive protein; IQR, interquartile range; SD indicates standard deviation; preop, preoperative; postop, postoperative; POD, postoperative day.

^a^ Three hundred and eighty‐one patients had POD1 CRP levels available for analysis.

^b^ Three hundred and fifty‐two patients had POD3 CRP levels available for analysis.

^c^ Three hundred and forty‐six patients had POD5 CRP levels available for analysis.

### 3.2 The prognostic value for CRPS during different periods

**FIGURE 1:**
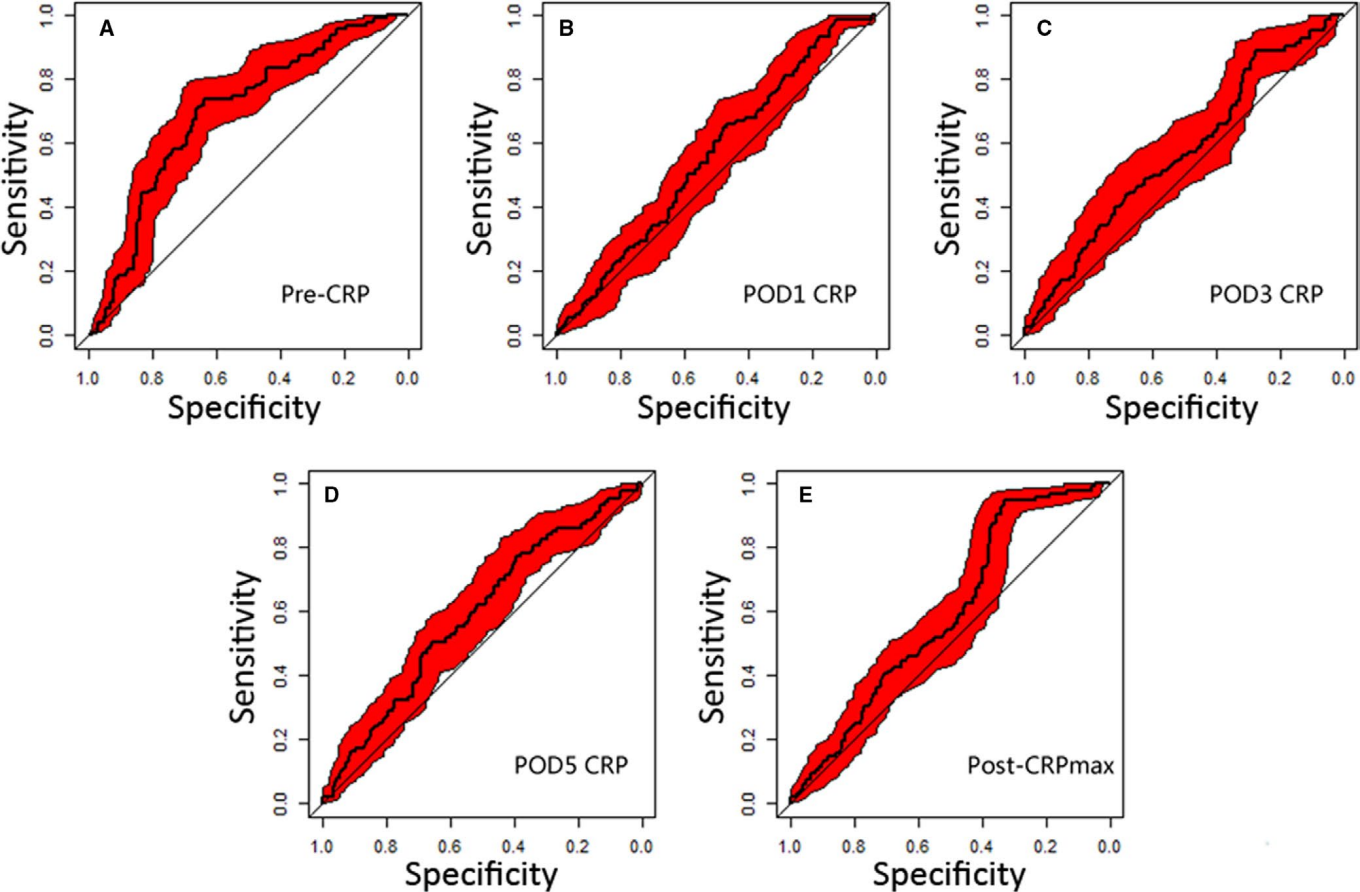
ROC curves for recurrence for CRPs at five different time points. Pre‐CRP indicates preoperative CRP; POD, postoperative day; Post‐CRP_max_, maximum value of postoperative CRP

Figure 1A‐E shows the ROC curves for five different CRPs, indicating that pre‐CRP had the highest AUC (0.692) compared with CRPs at different time periods (all *p* < 0.05). Similarly, pre‐CRP had the highest Concordance index (0.678). In addition, among the post‐CRPs, post‐CRP_max_ had the highest AUC (0.591) and Concordance index (0.585) (Table S2). Therefore, post‐CRP_max_ was selected to represent the post‐CRPs for subsequent analyses. The optimal cut‐off values for pre‐CRP and post‐CRP_max_ for RFS were 3.1 and 77.1 mg/L, respectively. According to these cut‐off values, patients were defined by the following categories: low‐pre‐CRP patients with pre‐CRP<3.1 mg/L, high‐pre‐CRP patients with pre‐CRP≥3.1 mg/L, low‐post‐CRP_max_ patients with post‐CRP_max_<77.1 mg/L, and high‐post‐CRP_max_ patients with post‐CRP_max_≥77.1 mg/L. Table S3 showed the association among complication status, preoperative CRP level, and postoperative CRP_max_ level. It was found that patients who experienced postoperative complication were more likely to be with high‐post‐CRP_max_ status.

### 3.3 Prognosis according to pre‐CRP and post‐CRP_max_ status

**FIGURE 2:**
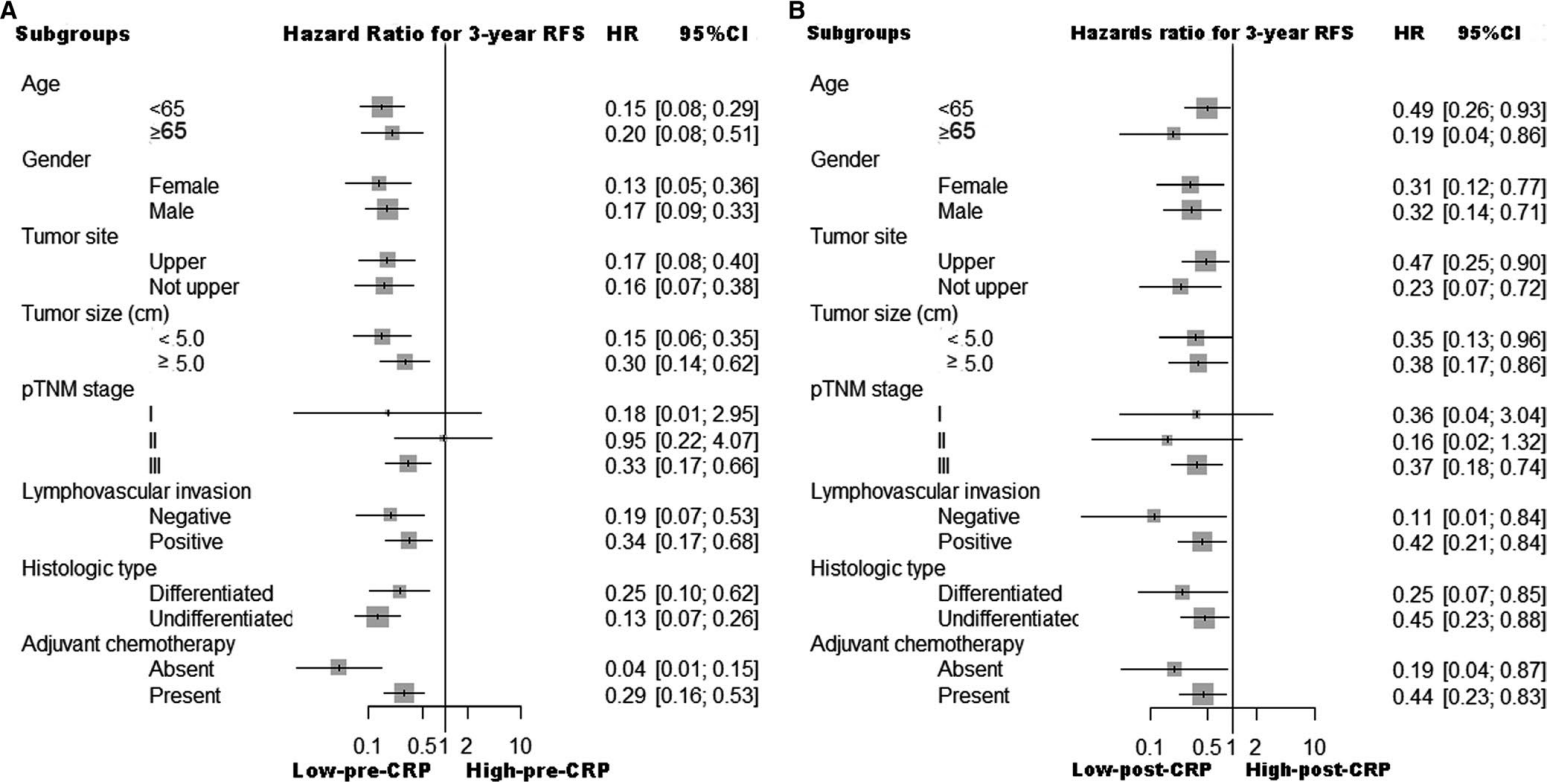
The relationship between (A) pre‐CRP, (B) post‐CRP_max_ status and 3‐year RFS in various subgroups

Overall, the prognoses of the high‐pre‐CRP group and the high‐post‐CRP_max_ group were significantly worse than the low‐pre‐CRP group or low‐post‐CRP_max_ group (3‐year RFS: 55.7% vs 87.8%, *p* < 0.001; 69.6% vs 79.8%, *p* = 0.041, respectively) (Figure S3A and S3B). In order to eliminate the potential effect of postoperative complication on prognosis, multivariate analysis which included postoperative complication showed that both pre‐CRP≥3.1 (HR: 1.728, 95% CI: 1.076‐2.733, *p* = 0.024) and post‐CRP_max_≥77.1 (HR: 1.631, 95% CI: 1.019‐2.611, *p* = 0.041) were independent risk factors of postoperative recurrence, and postoperative complication was not associated with postoperative recurrence. In addition, the interaction between postoperative complication and post‐CRP_max_ was not significant (*p* = 0.369), suggesting that postoperative complication did not influence the relationship between post‐CRP_max_ and RFS (Table 2). Furthermore, in a separate analysis of each clinicopathological factor, the prognostic value of the pre‐CRP and post‐CRP_max_ results was consistent (Figure 2A,B).

**TABLE 2:** Univariate and multivariate analyses of factors associated with recurrence‐free survival

	Univariate analysis			Multivariate analysis		
	HR	95%CI	*p* value	HR	95%CI	*p* value
Sex						
Female	1.000					
Male	1.248	0.823‐1.892	0.296			
Age (years)						
<65	1.000					
≥65	1.256	0.849‐1.858	0.254			
Tumor diameter (mm)						
<50	1.000			1.000		
≥50	4.433	2.954‐6.655	<0.001	1.460	0.947‐2.249	0.086
Tumor location						
Lower	1.000					
Middle	1.007	0.567‐1.787	0.982			
Upper	1.444	0.927‐2.249	0.104			
Mix	1.747	0.956‐3.194	0.070			
Pathological type						
Differentiated	1.000			1.000		
Undifferentiated	2.102	1.381‐3.201	0.001	1.086	0.704‐1.678	0.708
Lymphovascular invasion						
Absent	1.000			1.000		
Present	6.490	4.090‐10.298	<0.001	1.992	1.200‐3.308	0.008
Adjuvant chemotherapy						
Absent	1.000			1.000		
Present	2.285	1.442‐3.621	<0.001	0.525	0.319‐0.865	0.011
Preop CRP (mg/L)						
<3.1	1.000			1.000		
≥3.1	4.684	2.995‐7.327	<0.001	1.728	1.076‐2.773	0.024
Postop CRP_max_ (mg/L)						
<77.1	1.000			1.000		
≥77.1	1.606	1.014‐2.544	0.044	1.631	1.019‐2.611	0.041
Postop complication						
Absent	1.000			1.000		
Present	1.174	0.716‐1.926	0.525	1.105	0.659‐1.853	0.705
pTNM						
Ⅰ	1.000			1.000		
Ⅱ	3.825	0.989‐14.792	0.052	3.055	0.738‐12.654	0.124
Ⅲ	35.349	11.199‐111.578	<0.001	19.742	5.338‐73.006	<0.001
Interaction of postop complication with postop CRP_max_	—	—	—	—	—	0.369

Abbreviations: CRP, C‐reactive protein; CRP_max_, maximum CRP value; preop indicates preoperative; postop, postoperative.

**FIGURE 3:**
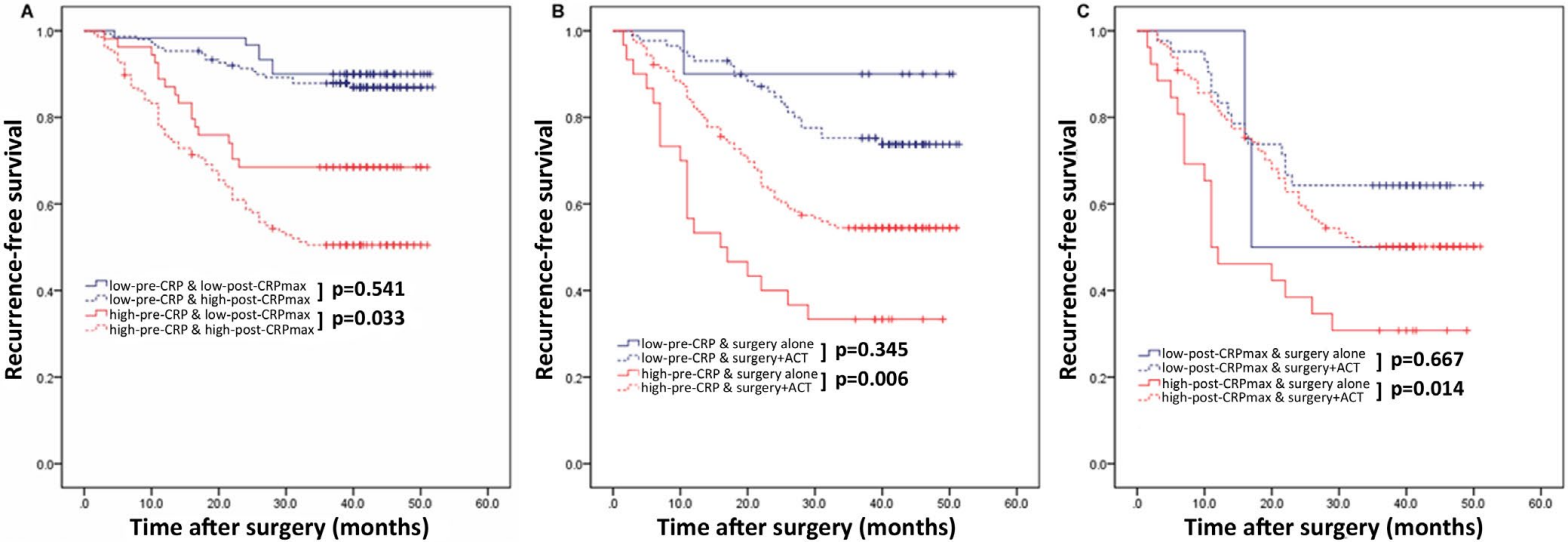
Comparison of recurrence‐free survival (A) in all GC patients according to combined pre‐CRP status and post‐CRP_max_ status; (B) in patients with stage II/III gastric cancer according to pre‐CRP status and treatment arm; and (C) in patients with stage II/III gastric cancer according to post‐CRP_max_ status and treatment arm for high‐pre‐CRP gastric cancer patients. ACT indicates adjuvant chemotherapy

Since both pre‐CRP and post‐CRP_max_ were closely associated with the prognosis, further survival analyses were evaluated according to combined pre‐CRP and post‐CRP_max_ status. As shown in Figure 3A, favorable prognosis was observed in the low‐pre‐CRP patients regardless of post‐CRP_max_ status (3‐year RFS: 90.0% [low‐post‐CRP_max_] vs 86.9% [high‐post‐CRP_max_], *p* = 0.541). In the high‐pre‐CRP group, however, low‐post‐CRP_max_ was related to a better prognosis than high‐post‐CRP_max_ (RFS: 68.5% vs 50.5%, *p* = 0.033).

### 3.4 Incorporation of pre‐CRP and post‐CRP_max_ levels into PTNM stage

By combining the pre‐CRP, post‐CRP_max_, and pTNM stage, a novel predictive model was established for RFS (model A). When compared with model B (comprising pTNM stage only), model A had a lower AIC (1123.7 vs 1131.6) and a higher Concordance index (0.814, 95% CI: 0.781‐0.848 vs 0.779, 95% CI: 0.749‐0.810, *p* = 0.003). When model A was subjected to 1000 bootstrap resamples for internal validation, it still showed excellent predictive value (corrected Concordance index: 0.809).

### 3.5 Clinical utility of the predictive model consisting of PTNM stage and pre‐CRP and post‐CRP_max_ levels

When we compared the net benefit of model A (comprising pre‐CRP, post‐CRP_max_, and pTNM stage) with model B, it was observed that, in a wide range of threshold probabilities (33% ~83%), the clinical net benefit of the former was greater than the latter. (Figure S4).

### 3.6 Effects of pre‐crp and post‐CRP_max_ on adjuvant chemotherapy

Table S4 showed the clinicopathological characteristics of the two groups according to whether they received ACT in stage II/III patients. Non‐chemotherapy group had more men [17 (42.5%) vs 60 (26.5%), *p* = 0.040], and was older (63.0 years vs 58.5 years, *p* = 0.007). No difference was observed between the two groups in tumor location, tumor diameter, pathological type, lymphovascular invasion, and postoperative complication, all *p* > 0.05. Further ACT benefit analyses showed that benefit from ACT was not clear in the low‐pre‐CRP group (3‐year RFS: 73.8% vs 90.0% in chemotherapy and surgery only groups, respectively, *p* = 0.345; 3‐year OS: 77.1% vs 80.0% in chemotherapy and surgery only groups, respectively, *p* = 0.964). In contrast, the outcome of patients receiving ACT was significantly improved only in the high‐pre‐CRP group (3‐year RFS: 54.5% vs 33.3%, *p* = 0.006; 3‐year OS: 51.8% vs 24.4%, *p* = 0.001) (Figure 3B, Figure S5A).

Because the prognosis of high‐pre‐CRP GC differed by post‐CRP_max_ status, we evaluated whether the benefit of ACT also differed according to post‐CRP_max_ status in high‐pre‐CRP GC. As Figure 3C and Figure S5B show, a significant improved prognosis from ACT was observed when compared with surgery only in the high‐post‐CRP_max_ subgroup of high‐pre‐CRP GC (3‐year RFS: 50.1% vs 30.8% in chemotherapy and surgery only groups, respectively; *p* = 0.014; 3‐year OS: 47.0% vs 23.1% in chemotherapy and surgery only groups, respectively; *p* = 0.003). However, there was no significant difference in the low‐post‐CRP_max_ subgroup according to the reception of ACT (3‐year RFS: 64.3% vs 50.0% in chemotherapy and surgery only groups, respectively, *p* = 0.667; 3‐year OS: 63.8% vs 50.8% in chemotherapy and surgery only groups, respectively, *p* = 0.636). Therefore, we established a suggested treatment algorithm for stage II/III GC after R0 resection according to pre‐CRP and post‐CRP_max_ statuses (Figure S6).

## 4 DISCUSSION

We present the post hoc analyses of a clinical trial and showed that both pre‐CRP and post‐CRP_max_ were closely associated with the recurrence for GC after R0 resection, and the predictive value of pre‐CRP was significantly greater than post‐CRPs. Compared with pTNM stage alone, incorporation of pre‐CRP and post‐CRP_max_ levels with pTNM stage significantly improved the predictive ability and clinical utility of the predictive model. Furthermore, our analyses for stage II/III GC showed that patients with pre‐CRP<3.1 had good prognosis and received unclear benefits from ACT. However, ACT should be focused on patients with pre‐CRP≥3.1.

Since the correlation between inflammation and cancer was uncovered,^9^ increasingly more evidence has shown that tumor progression is not only associated with the intrinsic properties of the cancer cells but is also related to the inflammatory immune response.^24^ Several studies confirmed that inflammatory indices, such as neutrophil‐lymphocyte ratio, CRP‐albumin ratio, and CRP‐prealbumin ratio, were closely associated with the prognosis of GC.^25^, ^26^, ^27^ However, study focusing on the prognostic value of combined pre‐CRP and post‐CRP for GC recurrence has not been reported.

CRP is mainly produced by liver cells and, to a lesser degree, by kidney, monocytes, and neutrophils.^28^ The rapid increase in serum concentration is associated with IL‐6, TNF‐α, and other proinflammatory cytokines.^29^ These proinflammatory cytokines accelerate angiogenesis, which in turn enhances the progression and metastasis of malignant tumors.^11^, ^30^ Cancer cells could also produce several cytokines and chemokines, thus leading to inflammatory cell infiltration into the tumor microenvironment (TME) and increasing the serum CRP concentration.^31^ Therefore, pre‐CRP level can reflect the TME status and its relationship with host immunity. In addition, CRP binds to the surface of apoptotic cells and activates the classic complement pathway, enhancing opsonization and phagocytosis of CRP‐tagged targets.^32^, ^33^ CRP can also recruit C4b‐binding protein, the main inhibitor of the classic complement pathway, and regulates the activity of immune cells, such as macrophages, neutrophils, and monocytes.^28^ Therefore, CRP may also be considered a regulator of innate immunity, rather than merely an indicator of inflammation.

Recently, serum CRP level has been shown to be closely related to the prognosis of a variety of malignant tumors, including breast cancer, colorectal cancer, and thymic epithelial tumors.^34^, ^35^, ^36^, ^37^ However, most study only focused on CRP levels before treatment. Few reports explored the relationship between posttreatment CRP levels and long‐term outcomes in patients with cancer. Pastorino et al. found for the first time that baseline and postoperative CRP levels were closely related to prognosis in patients with resectable lung cancer.^13^ At present, no study reported the influence of perioperative CRP levels on the prognosis of patients with GC. For the first time, the present study demonstrated that both pre‐CRP level and post‐CRP_max_ were closely related to postoperative recurrence of GC by multivariate analysis.

By constructing ROC curves and calculating the AUC and C‐index, we found an interesting phenomenon that pre‐CRP had a significantly higher predictive value than post‐CRP_max_ for GC recurrence. The reason for this finding may be that post‐CRP levels could be affected by surgical stress and complications.^38^, ^39^ As a result, although post‐CRP_max_ has independent prognostic value for prognosis, there could be additional confounding factors beyond pre‐CRP. In addition, previous study showed that postoperative complication had a negative effect on recurrence and prognosis for GC patients.^40^, ^41^ However, in the present study, although the postoperative complication is related to the high‐post‐CRP_max_, it failed to affect the postoperative recurrence. Further interaction analysis suggested that postoperative complication did not influence the relationship between post‐CRP_max_ and RFS. Research conducted by Saito showed the similar results.^42^ These findings suggested that the post‐CRP_max_ level in the early postoperative phase may be helpful for the postoperative complication and for the prediction of long‐term prognosis. Further study is needed to validate the results.

To further evaluate the predictive value of pre‐CRP and post‐CRP for the prognosis of gastric cancer (GC), we constructed a predictive model, model A, including pre‐CRP, post‐CRP_max_, and pTNM stage. The C‐index was significantly higher, and the AIC value was lower than model B (pTNM stage only). Since the decision curve analysis was proposed in 2006,^23^ it has been widely accepted by scholars to calculate net benefits and evaluate the clinical utility of predictive models, including the utility of models for cancer patients.^43^, ^44^, ^45^, ^46^ The DCA further confirmed the clinical utility of model A.

Numerous studies have demonstrated that ACT can significantly improve the RFS^6^, ^7^, ^8^ for GC. However, it is also essential for doctors to identify patients who could benefit from ACT. At present, several scholars have carried out relevant research. Sohn found that GC with chromosomal instability molecular subtypes could benefit from ACT.^47^ Cheong et al. constructed a single patient classifier to screen out GC patients who benefited from ACT^48^ Ji and Young et al. found that microsatellite instability was related to the efficacy of ACT.^49^, ^50^ Compared with the above results, CRP, an inflammation‐based marker, has been routinely tested in clinic, and it is cheaper and easier to obtain. Based on the present findings, we recommend using pre‐CRP status and post‐CRP_max_ status for risk stratification and deciding on the appropriateness of ACT for patients with stage II/III GC (Figure S6). For GC patients with low‐pre‐CRP, regardless of their post‐CRP_max_ level, we suspected that ACT after radical gastrectomy might not be an optimal strategy due to this form of GC having a favorable prognosis and an unclear benefit from ACT. However, due to the small sample size, this hypothesis needs to be validated in more larger cohorts in future. For high‐pre‐CRP patients with high‐post‐CRP_max_ status, the prognosis was poor, but the benefit of ACT was obvious. Therefore, ACT should be strongly recommended for this subgroup. Although the survival of high‐pre‐CRP GC with low‐post‐CRP_max_ was better than that of high‐pre‐CRP GC with high‐post‐CRP_max_, it was still worse than low‐pre‐CRP GC, while no benefit from ACT was observed.

Therefore, in order to further improve the prognosis of this subgroup, a large sample of clinical trials is warranted for this particular subgroup to evaluate the potential use of different ACT regimens. What is more, according to previous reports, dynamic changes in inflammatory markers, such as NLR, and tumor markers, such as CA19‐9, before and after neoadjuvant chemotherapy are also associated with long‐term prognosis of patients with tumors.^51^, ^52^, ^53^, ^54^ Similarly, future clinical trials can be conducted to further assess whether high‐pre‐CRP GC can benefit from neoadjuvant chemotherapy and to assess the predictive value of the dynamic change in CRP levels before and after neoadjuvant chemotherapy, which would assist clinicians in formulating the best treatment options for such patients.

Considering previous evidence that statins or metformin can reduce serum CRP levels,^55^, ^56^ our findings open new prospects for ACT in GC after radical gastrectomy. CRP levels could be used to determine the efficacy of anti‐inflammatory drugs, such as COX‐2 inhibitors, metformin, or statins,^57^ in randomized clinical trials to improve the long‐term prognosis for GC. Prospective trials could further explore the CRP as a reliable intermediate biomarker to predict the long‐term effects of such drug interventions. In addition, we hope to confirm our findings through further prospective clinical trials. Further basic studies will be conducted to determine how CRP participates in the progress of GC and how CRP affects the efficacy of ACT.

Recently, several studies showed that serum amyloid A (SAA) before treatment is associated with the prognosis of patients with solid tumors, including GC.^58^, ^59^ Due to the inherent defects of retrospective study, SAA levels were not detected in this cohorts. Therefore, we were unable to explore the relationship between SAA and CRP levels and long‐term outcomes in patients with GC. Further large studies are warranted in the future to explore the correlation among SAA and CRP levels and prognosis of patients with GC.

This study had several potential drawbacks. First, it was a monocentric exploratory study without external validation, and the sample size was small. Second, due to the small sample size, we did not analyze that whether the detailed cycles and regimens of ACT altered the relationship between CRP levels and prognosis. Third, the median follow‐up of 41 months was relatively short. However, when it occurs, recurrence of GC usually develops within the first 2 years after surgery,^5^, ^60^ so it may be sufficient considering the recurrence patterns in the present study. As far as we know, this study is the first to combine pre‐CRP and post‐CRP levels and investigate their relationship with postoperative recurrence and the efficacy of ACT, which provides a basis and direction for further clinical trials in the future.

In conclusion, through post hoc analysis of a prospective clinical trial, we demonstrated that pre‐CRP and post‐CRP_max_ statuses were clinically actionable as potential prognostic indicators and could be used as supplements to traditional pTNM staging. In addition, pre‐CRP and post‐CRP_max_ statuses were helpful to optimize the treatment decision for GC. For stage II/III GC patients with pre‐CRP ≥3.1 mg/L and post‐CRP_max_ ≥77.1 mg/L, ACT should be strongly recommended. Further multicenter validation studies are warranted.

## DISCLOSURE

There are no conflict of interest or financial ties to disclose from any authors.

## AUTHOR CONTRIBUTIONS

Jun Lu, Bin‐bin Xu, and Ping Li conceived the study, analyzed the data, and drafted the manuscript; Zhen Xue, Jian‐wei Xie, Chao‐hui Zheng, and Chang‐ming Huang helped critically revise the manuscript for important intellectual content; Jun Lu, Bin‐bin Xu, Chao‐hui Zheng, Chang‐ming Huang, and Ping Li helped collect data and design the study.

## Supporting information

Fig S1

Fig S2

Fig S3

Fig S4

Fig S5

Fig S6

Table S1

Table S2

Table S3

Table S4

## Data Availability

The dataset analyzed for this study is available from the corresponding author on reasonable request.
