# Supplementary figures and images for: Perioperative CRP: A novel inflammation‐based classification in gastric cancer for recurrence and chemotherapy benefit
Source: Cancer Med. 2020 Dec 3;10(1):34–44. doi: 10.1002/cam4.3514 (PMC7826470; doi:10.1002/cam4.3514)

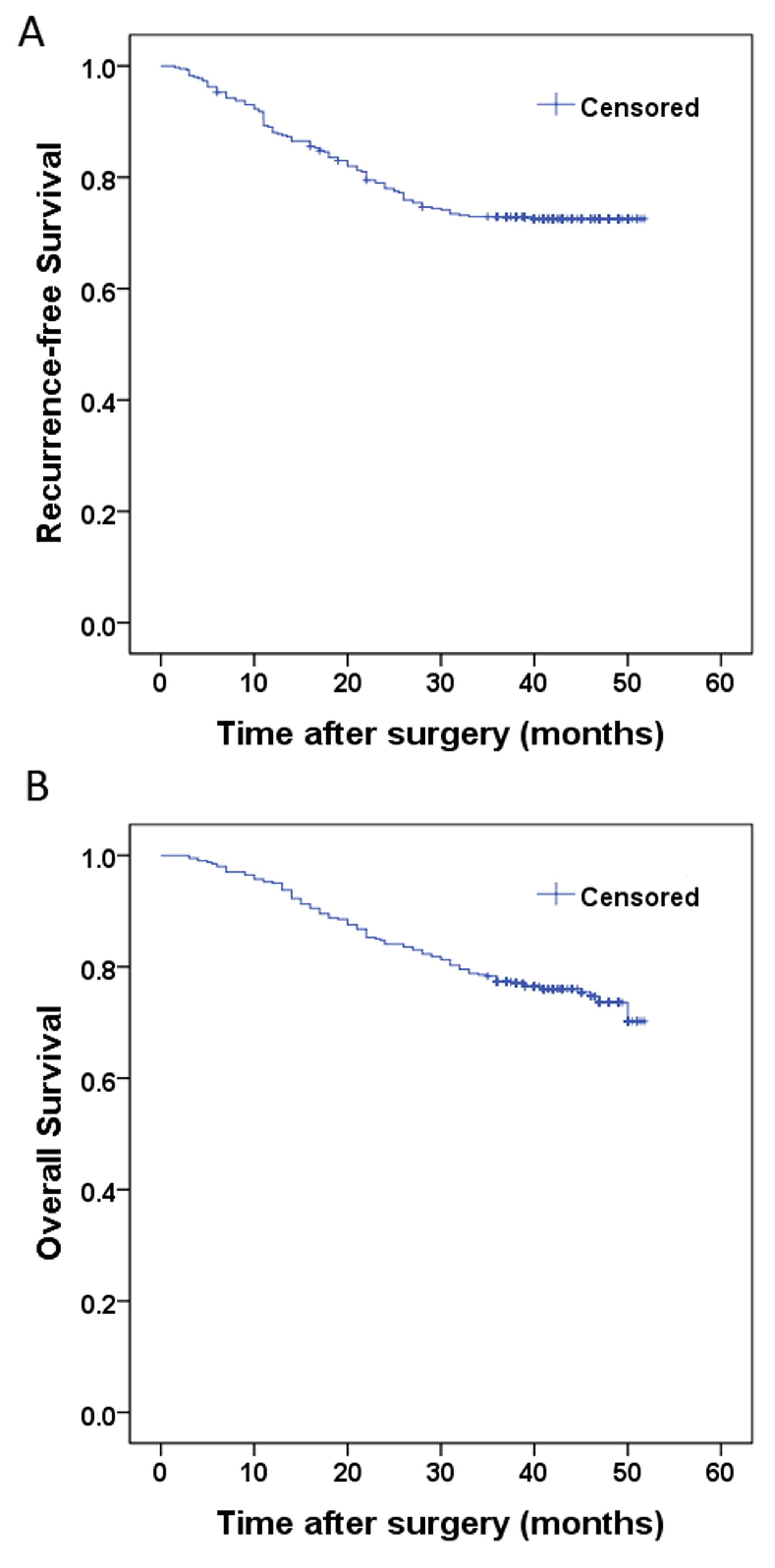

Supplement: Supplementary file 1 — Fig S1 [file CAM4-10-34-s001.tif]

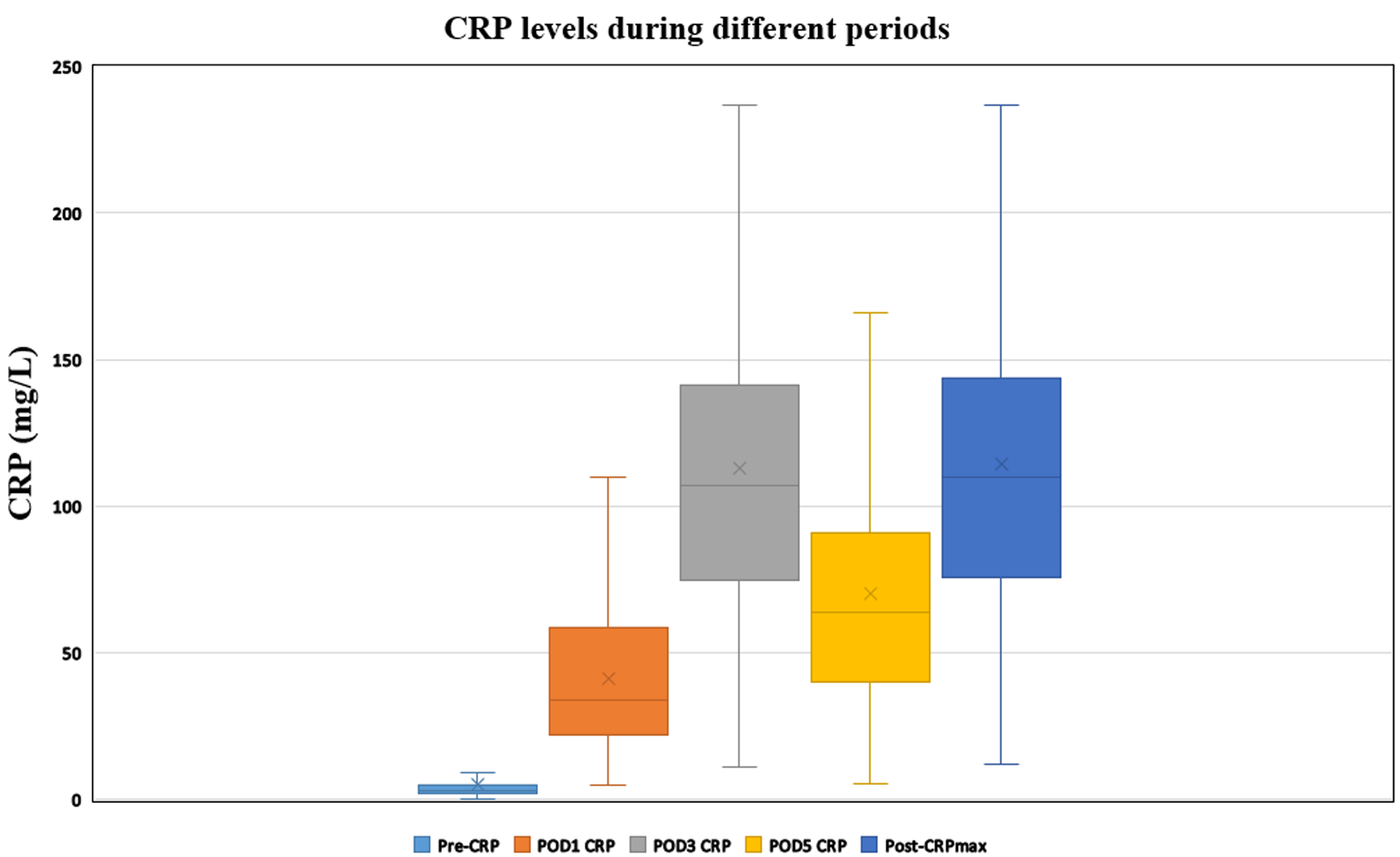

Supplement: Supplementary file 2 — Fig S2 [file CAM4-10-34-s002.tif]

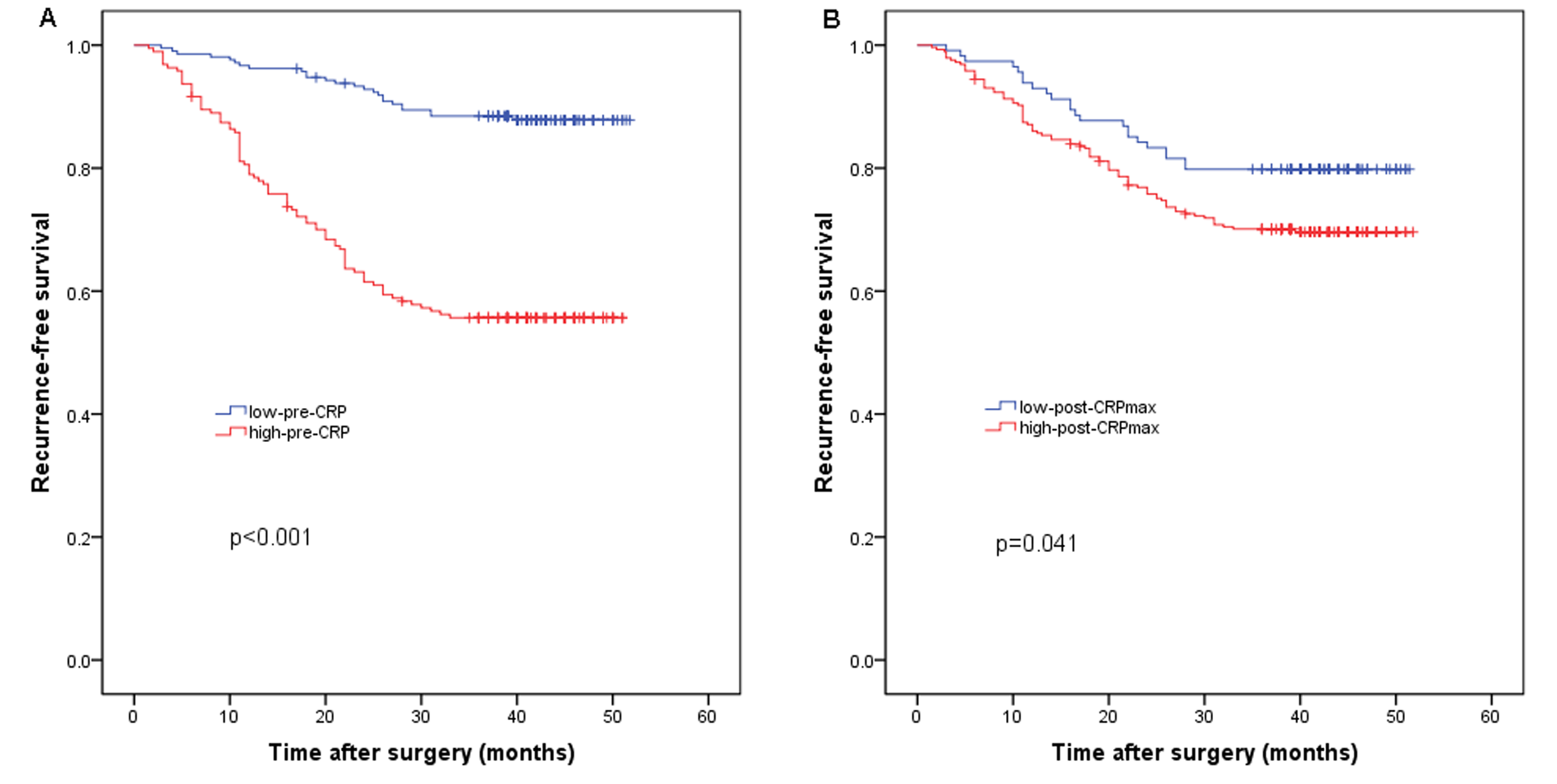

Supplement: Supplementary file 3 — Fig S3 [file CAM4-10-34-s003.tif]

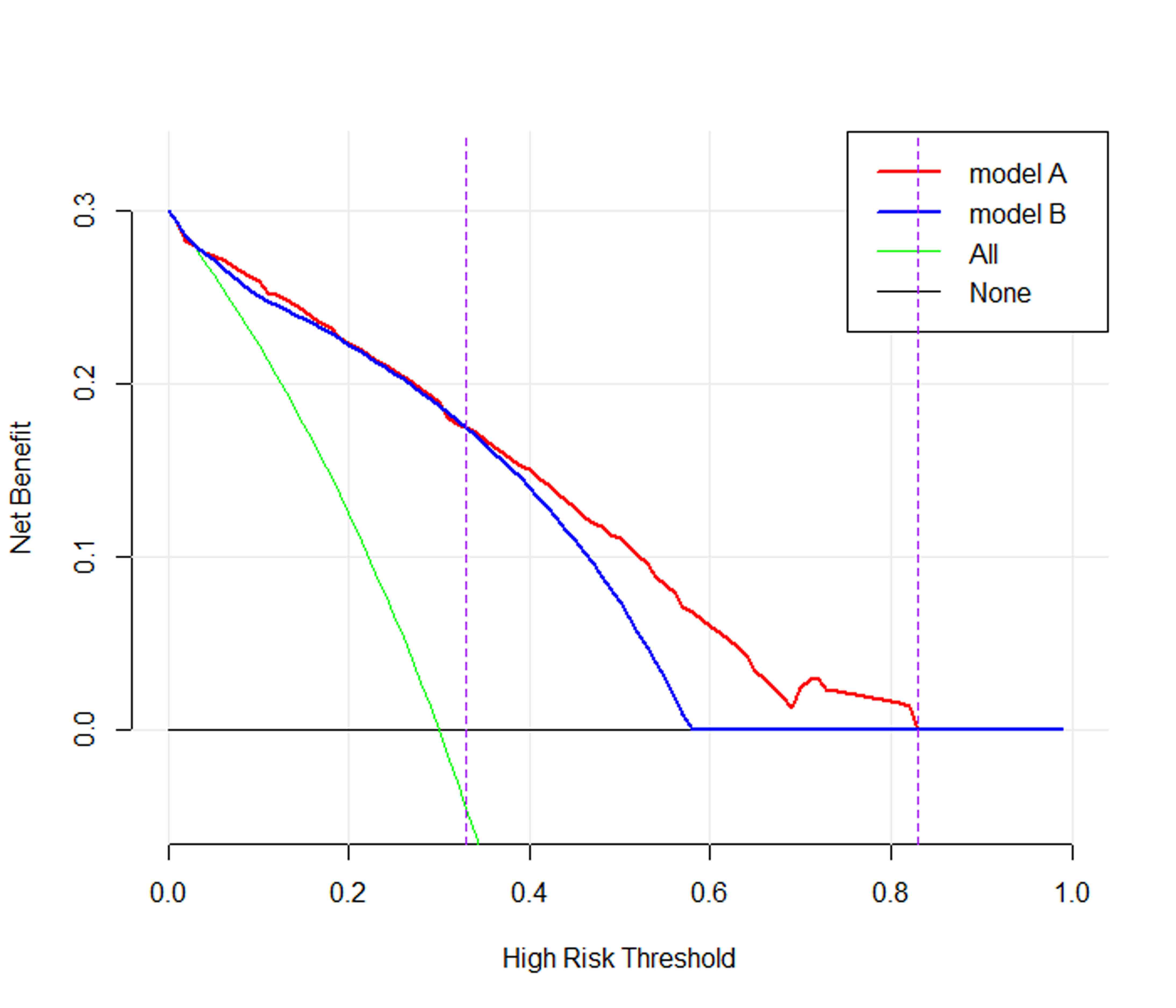

Supplement: Supplementary file 4 — Fig S4 [file CAM4-10-34-s004.tif]

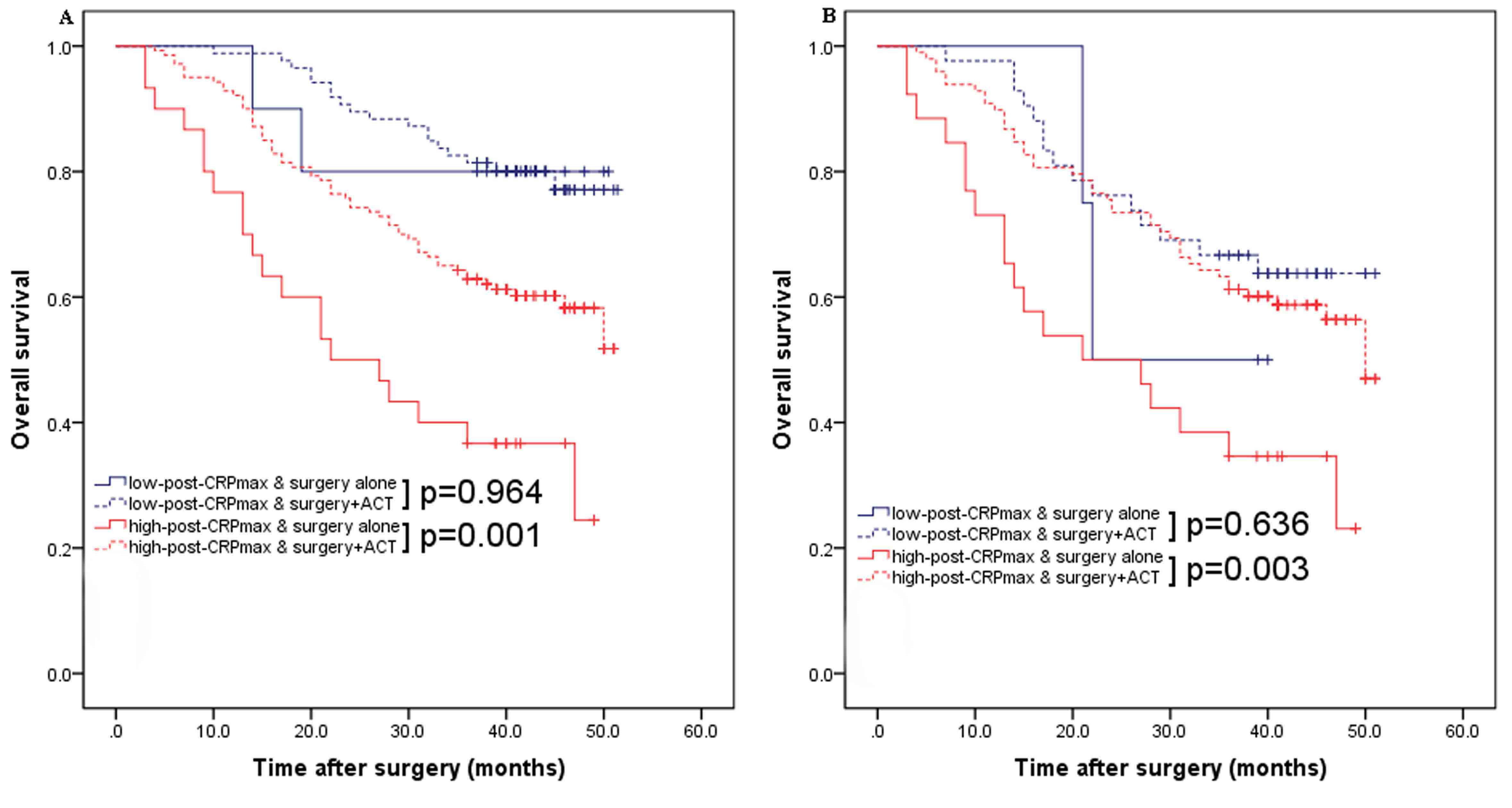

Supplement: Supplementary file 5 — Fig S5 [file CAM4-10-34-s005.tif]

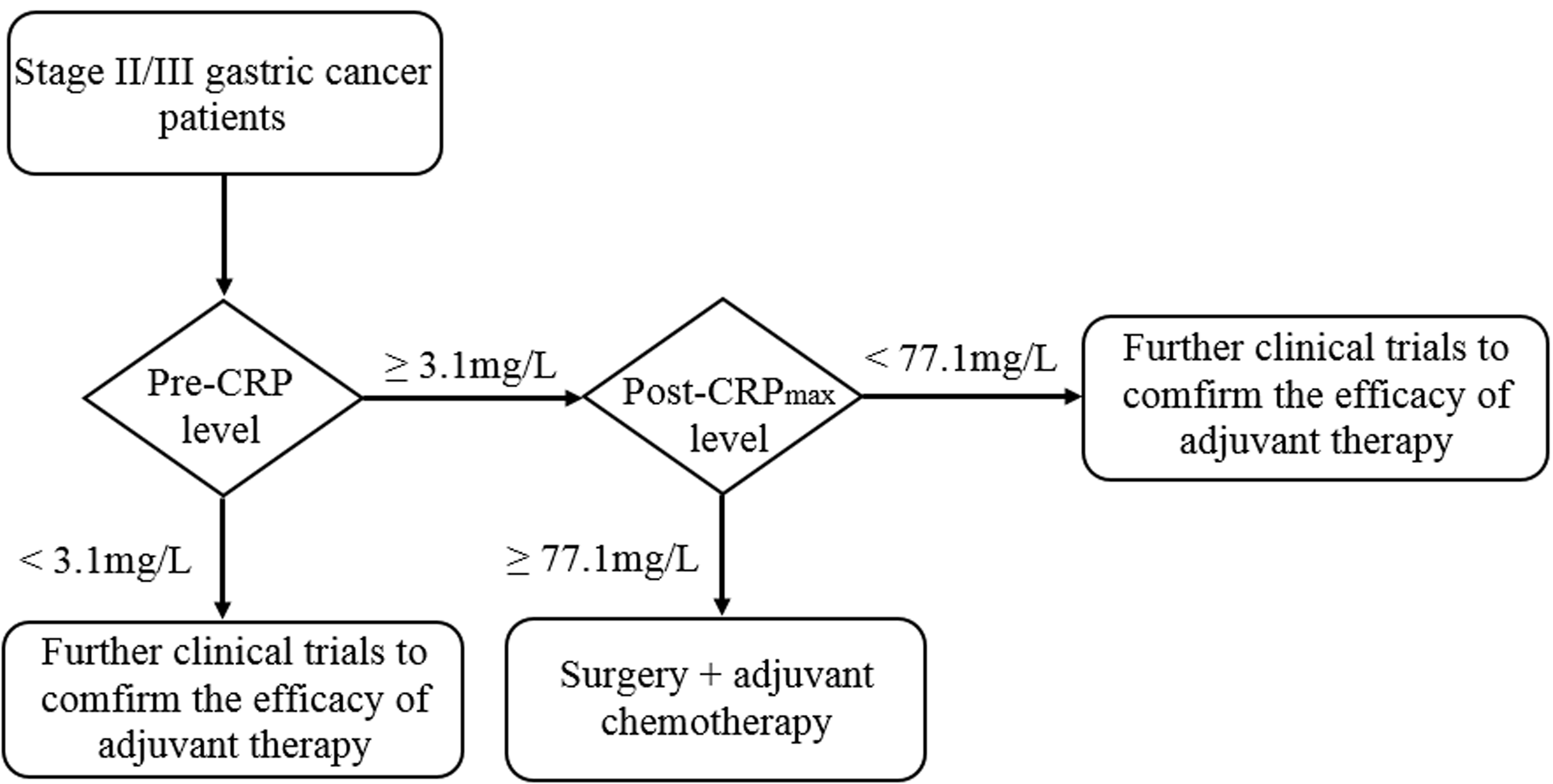

Supplement: Supplementary file 6 — Fig S6 [file CAM4-10-34-s006.tif]
