# Supplementary material for: Perioperative CRP: A novel inflammation‐based classification in gastric cancer for recurrence and chemotherapy benefit
Source: Cancer Med. 2020 Dec 3;10(1):34–44. doi: 10.1002/cam4.3514 (PMC7826470; doi:10.1002/cam4.3514)
Supplement: Supplementary file 7 — Table S1 [file CAM4-10-34-s007.docx]

**Table S1. The detailed inclusion and exclusion criteria for the primary clinical trial**

| **Inclusion Criteria** |
| --- |
| (1) Age from over 19 to under 74 years  (2) cT1-4a (clinical stage tumor), N0-3, M0 at preoperative evaluation according to the American Joint Committee on Cancer (AJCC) Cancer Staging Manual Seventh Edition  (3) Heart, lungs, kidneys and other vital organs function well, with no obvious surgical contraindications  (4) Preoperative examination with no distant metastasis, no significantly enlarged lymph nodes around abdominal main artery, and tumor not a direct violation of the pancreas, spleen and other surrounding organs  (5) American Society of Anesthesiology (ASA) score class I, II, or III; (6) Written informed consent; |
| **Exclusion Criteria** |
| (1) Women during pregnancy or breast-feeding  (2) Severe mental disorder  (3) History of previous upper abdominal surgery (except laparoscopic cholecystectomy)  (4) Enlarged splenic hilar lymph nodes with integration into a mass and surrounding the blood vessels  (5) History of unstable angina or myocardial infarction within past six months  (6) History of cerebrovascular accident within past six months  (7) History of continuous systematic administration of corticosteroids within one month  (8) History of previous neoadjuvant chemotherapy or radiotherapy  (9) T4b tumors  (10) Emergency surgery due to complication (bleeding, obstruction or perforation) caused by gastric cancer  (11) FEV1(Forced expiratory volume in one second)＜50% of predicted values. |
