# Supplementary material for: Perioperative CRP: A novel inflammation‐based classification in gastric cancer for recurrence and chemotherapy benefit
Source: Cancer Med. 2020 Dec 3;10(1):34–44. doi: 10.1002/cam4.3514 (PMC7826470; doi:10.1002/cam4.3514)
Supplement: Supplementary file 8 — Table S2 [file CAM4-10-34-s008.docx]

| **Table S2 Comparison of the AUC and C-index among the CRP levels detected in different periods.** | | | | | | |
| --- | --- | --- | --- | --- | --- | --- |
|  |  | |  |  |  |  |
| Periods | AUC | 95%CI | p value * | C-index | 95%CI | p value† |
| Preoperative CRP | 0.692 | 0.634-0.750 | - | 0.678 | 0.627-0.729 | - |
| POD1 CRP | 0.558 | 0.494-0.623 | 0.002 | 0.556 | 0.500-0.612 | <0.001 |
| POD3 CRP | 0.569 | 0.499-0.639 | 0.01 | 0.566 | 0.503-0.628 | <0.001 |
| POD5 CRP | 0.588 | 0.520-0.656 | 0.009 | 0.577 | 0.517-0.637 | 0.005 |
| Postoperative CRPmax | 0.591 | 0.531-0.651 | 0.013 | 0.585 | 0.532-0.638 | 0.013 |
| *Comparison of AUC values between the preoperative CRP and other periods CRP was using Delong’s test. | | | | | | |
| †Comparison of C-index values between the preoperative CRP and other periods CRP. | | | | | | |
