# Supplementary material for: Perioperative CRP: A novel inflammation‐based classification in gastric cancer for recurrence and chemotherapy benefit
Source: Cancer Med. 2020 Dec 3;10(1):34–44. doi: 10.1002/cam4.3514 (PMC7826470; doi:10.1002/cam4.3514)
Supplement: Supplementary file 9 — Table S3 [file CAM4-10-34-s009.docx]

| **Table S3 Associations among complication status, preoperative CRP level and postoperative CRP_max_ level.** | | | | | | |
| --- | --- | --- | --- | --- | --- | --- |
|  |  | | |  | | |
|  | Postop complication (-) (n=339) | Postop complication (+) (n=62) | p value | Post CRP_max_ < 77.1 mg/L (n=114) | Post CRP_max_ ≥ 77.1 mg/L (n=287) | p value |
| Preop CRP (mg/L) |  |  | 0.216 |  |  | 0.947 |
| < 3.1 | 182 (53.7%) | 28 (45.2%) |  | 60 (52.6%) | 150 (52.3%) |  |
| ≥3.1 | 157 (46.3%) | 34 (54.8%) |  | 54 (47.4%) | 137 (47.7%) |  |
| Postop CRP_max_ (mg/L) |  |  | 0.020 |  |  | NA |
| < 77.1 | 104 (30.7%) | 10 (16.1%) |  | NA | NA |  |
| ≥77.1 | 235 (69.3%) | 52 (83.9%) |  | NA | NA |  |
| preop indicates preoperative; postop, postoperative; CRP, C-reactive protein; CRPmax, maximum CRP value; NA, not applicable. | | | | | | |
