# Supplementary material for: Perioperative CRP: A novel inflammation‐based classification in gastric cancer for recurrence and chemotherapy benefit
Source: Cancer Med. 2020 Dec 3;10(1):34–44. doi: 10.1002/cam4.3514 (PMC7826470; doi:10.1002/cam4.3514)
Supplement: Supplementary file 10 — Table S4 [file CAM4-10-34-s010.docx]

| **Table S4 Clinicopathological characteristics of stage II/III patients** | | | |
| --- | --- | --- | --- |
|  | Adjuvant chemotherapy | | |
|  | Absent (n=40) | Present (n=226) | p value |
| Age, mean years (SD) | 63.0 (11.1) | 58.5 (9.3) | 0.007 |
| Sex n (%) |  |  | 0.040 |
| Male | 17 (42.5%) | 60 (26.5%) |  |
| Female | 23 (57.5%) | 166 (73.5%) |  |
| Tumor location n (%) |  |  | 0.145 |
| Upper | 17 (42.5%) | 82 (36.3%) |  |
| Middle | 11 (27.5%) | 36 (15.9%) |  |
| Lower | 10 (25.0%) | 94 (41.6%) |  |
| Mix | 2 (5.0%) | 14 (6.2%) |  |
| Tumor diameter (mm) |  |  | 0.148 |
| <50 | 14 (35.0%) | 107 (47.3%) |  |
| ≥50 | 26 (65.0%) | 119 (52.7%) |  |
| Pathological type n (%) |  |  | 0.543 |
| Differentiated | 12 (30.0%) | 79 (35.0%) |  |
| Undifferentiated | 28 (70.0%) | 147 (65.0%) |  |
| Lymphovascular invasion n (%) |  |  | 0.421 |
| Absent | 17 (42.5%) | 81 (35.8%) |  |
| Present | 23 (57.5%) | 145 (64.2%) |  |
| Postoperative complication n (%) |  |  | 0.419 |
| Absent | 35 (87.5%) | 186 (82.3%) |  |
| Present | 5 (12.5%) | 40 (17.7%) |  |
